# Influence of Spirituality on Depression, Posttraumatic Stress Disorder, and Suicidality in Active Duty Military Personnel

**DOI:** 10.1155/2012/425463

**Published:** 2012-06-20

**Authors:** Laurel L. Hourani, Jason Williams, Valerie Forman-Hoffman, Marian E. Lane, Belinda Weimer, Robert M. Bray

**Affiliations:** Behavioral Health and Criminal Justice Division, RTI International, 3040 Cornwallis Road, P.O. Box 12194, Research Triangle Park, NC 27709, USA

## Abstract

Understanding the role of spirituality as a potential coping mechanism for military personnel is important given growing concern about the mental health issues of personnel returning from war. This study seeks to determine the extent to which spirituality is associated with selected mental health problems among active duty military personnel and whether it moderates the relationship between combat exposure/deployment and (a) depression, (b) posttraumatic stress disorder (PTSD), and (c) suicidality in active duty military personnel. Data were drawn from the 2008 Department of Defense Survey of Health Related Behaviors Among Active Duty Military Personnel. Over 24,000 randomly selected active duty personnel worldwide completed an anonymous self-report questionnaire. High spirituality had a significant protective effect only for depression symptoms. Medium, as opposed to high or low, levels of spirituality buffered each of the mental health outcomes to some degree. Medium and low spirituality levels predicted depression symptoms but only among those with moderate combat exposure. Medium spirituality levels also predicted PTSD symptoms among those with moderate levels of combat exposure and predicted self-reported suicidal ideation/attempt among those never deployed. These results point to the complex relationship between spirituality and mental health, particularly among military personnel and the need for further research.

## 1. Introduction

A sizable amount of literature has focused on the influence of religiosity and spirituality on health outcomes. Many of these studies are summarized in the 2001 book: *Handbook of Religion and Health* [1] and in Koenig et al. [2]. Research has shown associations between both concepts of spirituality and religiosity and mental health. For example, studies have indicated a significant relationship between higher levels of religiosity and lower levels of depression [3, 4], particularly among those at high risk for depression [5]. The association between higher levels of spirituality and lower levels of depressive symptoms has been demonstrated in several different subpopulations, including adolescent girls [6], patients in an urban clinic [7], and terminally ill patients with cancer and AIDS [8]. In addition, findings from the Baltimore Epidemiologic Catchment Area Study indicated that individuals who reported ever seeking spiritual comfort at Wave 3 assessment had decreased odds of suicidal ideation at Wave 4 as compared to those who reported never seeking spiritual comfort at Wave 3 [9]. The literature examining the relationship between spirituality, religion, and anxiety disorders and suicide is less robust [10]. However, greater levels of spirituality have been associated with fewer anxiety symptoms among patients with advanced illness [11] and with lower posttraumatic symptom severity among survivors of violent trauma [12]. Krejci et al. [13] found that although sexual abuse victims and controls did not differ in terms of spiritual well-being, lower psychopathology including PTSD symptom scores, were associated with increased spiritual well-being in both groups.

Several hypotheses have emerged that explain spirituality's link with mental disorders. Dating back to the end of the 19th century, Durkheim's study of religiosity/spirituality and suicidal behaviors suggested that those with increased spirituality also had greater levels of social support, which effectively buffered against psychopathology and suicidal behaviors [14]. More recent studies have come to similar conclusions [2, 3]. Another mechanism may be that greater spirituality protects against mental disorders and/or suicide by increasing the ability to cope with stressors [15], deepening one's sense of purpose or meaning [16], and/or reducing feelings of hopelessness, which have been indicated as a predictor of suicide [17]. In contrast, it has been suggested that religious beliefs may exert a negative influence on mental health especially in the emotionally vulnerable by increasing fears or guilt, reinforcing neurotic tendencies [2].

There have been a few published studies investigating the relationship between spirituality/religiosity and mental disorders and suicide in veteran samples. In a recently published study by Berg [18], spiritual distress was related to both combat-related posttraumatic stress disorder (PTSD) and depression in a sample of Vietnam veterans. Subjective religiosity (the extent religious beliefs are a source of comfort/strength) was shown to be associated with both women veterans [19] and male veterans [20] with sexual assault experience. Spiritual well-being was also found to be a mediator of the effect of a mantra (a sacred word or phrase repeated over and over) intervention to decrease PTSD symptoms in veterans with military trauma [21]. Other studies have examined interventions that include a spirituality component to help promote resilience (Pargament and Sweeney [22]), reduce health risks (Niederhauser et al. [23]), integrate health promotion and wellness (Parker et al. [24]), and reduce PTSD symptoms in veterans exposed to trauma (Harris et al. [25]), though all indicate that further, more robust research is warranted in these areas.

Additional investigations in military populations are needed given the potential importance of spirituality in helping active duty service members cope with deployment as well as transition from deployed to nondeployed status during wartime. Although programs such as the Warrior Transition Units have been created, they are not currently able to fully meet the needs of all returning service members [26]. Thus, the role of spirituality as a coping mechanism in the transition from deployed to nondeployed status and various associated stressors from recent combat experience may be important [27]. Spiritual fitness was named as one of the eight domains comprising a new framework of Total Force Fitness, an initiative from the Chairman of the Joint Chiefs of Staff for maintaining the health, readiness, and performance of Department of Defense (DoD) service members [28]. As part of the Army's Comprehensive Soldier Program which is comprised of a spiritual fitness tracker that requires soldiers to respond to statements such as, “I am a spiritual person, my life has lasting meaning, I believe that in some way my life is closely connected to all humanity and all the world,” this program has come under recent controversy [29, 30]. The recommendations call for the development of evidence-based spiritual support policies and programs and integrating chaplains as primary providers of such services [31]. In 2010, DoD and the Department of Veterans Affairs (VA) launched a joint strategic action implementation plan under the DoD/VA Integrated Mental Health Strategy to increase understanding of chaplains' roles in primary care within the military treatment system and VA; to support continuity in the role of chaplains between the services and VA; to identify ways to facilitate access to mental health care through collaboration with community clergy and faith communities. Investigation of the relationship between spirituality and mental health problems is particularly important given that active duty members have particularly high rates of suicidal ideation and extreme anxiety as compared to reports from general population samples [32–34].

The present study seeks to address two research aims. The first aim is to determine the extent to which spirituality is associated with selected mental health problems (i.e., depression, PTSD, and suicidality) among active duty military personnel controlling for potential demographic, coping, and service-related variables. We hypothesize that high spirituality, defined as the self-reported importance of religious/spiritual beliefs in life and decision making, is protective against depression, PTSD, and suicidality. Although the measure used in this study combines both concepts of religiosity and spirituality, we refer to the broader concept of spirituality to encompass the combination of the concepts in this measure throughout this paper.

The second aim is to determine whether spirituality moderates the relationship between combat exposure/deployment and these mental health problems. We hypothesize that high levels of spirituality will buffer the association between combat exposure/deployment and depression, PTSD, and suicidality.

## 2. Materials and Methods

### 2.1. Subjects

Data were drawn from the 2008 DoD Survey of Health Related Behaviors Among Active Duty Military Personnel (2008 HRB Survey) [29]. The survey consists of a randomly selected representative sample of active duty military personnel from the Army, Navy, Marine Corps, Air Force, and Coast Guard. A two-stage replacement cluster sample proportional to size was employed in which geographic areas were clustered and randomly selected in the first stage, and individuals within the clusters were randomly selected in the second stage. All active duty members were eligible except for recruits, academy cadets, and persons who were absent without leave or incarcerated. The final sample of participants consisted of 28,546 military personnel (5,927 Army, 6,637 Navy, 5,117 Marine Corps, 7,009 Air Force, and 3,856 Coast Guard), who completed self-administered questionnaires anonymously. The overall response rate was 70.6%. Although the number of missing items tended to increase with the length of the questionnaire, the response rate was high relative to most military surveys, and most items showed less than a 5% missing rate. Data were weighted to represent all active duty personnel, meaning that the results of the survey represent population estimates of the entire active force. The current study included only the DoD services (Army, Navy, Air Force, and Marine Corps) for a study total of 24,690.

### 2.2. Survey Procedures

The majority (97%) of the 32-page anonymous self-report questionnaires were obtained during onsite visits to 64 military installations worldwide by the study team. The rest were obtained from questionnaires mailed to respondents who were unable to attend group sessions. At the group sessions, survey data collection field teams described the purpose of the study, assured participants of anonymity, informed participants of the voluntary nature of the survey, distributed introductory handouts, ensured that an ombudsperson was present for each group administration to attest that teams explained the voluntary nature of participation, and showed personnel the correct procedures for marking the questionnaire. Team members then distributed the optical-mark questionnaires to participants, who completed and returned them. On average, the questionnaire required about an hour to complete. Institutional Review Board approval was obtained from RTI International and DoD. Additional sampling and methodological details have been reported and published elsewhere [35].

### 2.3. Key Measures

#### 2.3.1. Demographics 

Demographics included gender (male and female) race/ethnicity (white non-Hispanic, black non-Hispanic, Hispanic, and other), highest level of education (high school diploma or less, some college, and college degree or more) age group (17–20, 21–25, 26–34, and 35 or older), and military pay grade (E1–E3 junior enlisted, E4–E6 middle enlisted, E7–E9 senior enlisted, W1–W5 warrant officer, O1–O3 junior commissioned officers, and O4–O10 middle and senior officers).

#### 2.3.2. Spirituality

Respondents were asked to what extent they agreed with two questions regarding the importance of religious/spiritual beliefs and the degree to which religious/spiritual beliefs influenced their decision making. Items were “My religious/spiritual beliefs are a very important part of my life” and “My religious/spiritual beliefs influence how I make decisions in my life,” each rated on a 4-point scale from strongly agree to strongly disagree. Respondents' spirituality was categorized as high if they reported “strongly agree” to both questions, moderate if they reported either “strongly agree” or “agree” to at least one of the questions, and low if they reported either “disagree” or “strongly disagree” to both questions. These items were adapted from those used in the National Survey on Drug Use and Health [36]. 

#### 2.3.3. Combat Exposure

Exposure to combat and related circumstances was measured using a modified version of the 17-item Combat Experiences Scale of the Deployment Risk and Resilience Inventory [37]. These items assess exposure to incoming fire, mines, improvised explosive devices, firing on the enemy, viewing dead bodies or human remains, interaction with enemy prisoners of war, and similar circumstances that may be relevant. Each item asked how many times the respondent was exposed, and response options were 0 (0 times), 1 (1 to 3 times), 2 (4 to 12 times), 3 (13 to 50 times), and 4 (51 or more times). All items were summed, and the sum score was used to create a categorical combat exposure item where a score equal to zero was considered “deployed but no exposure to combat,” a score from 1 to 9 was classified as “low/moderate combat exposure,” and a score of 10 or greater was considered as “moderate/high combat exposure.” A fourth category was added to capture personnel who had not been deployed. These cutoffs were subsequently examined with factor analysis and item scoring methods suggesting that these categories captured meaningful distinctions between groups of scores.

#### 2.3.4. Coping 

Respondents were asked to identify the types of strategies that they use to cope when they “feel pressured, stressed, depressed, or anxious.” The list of strategies included items that assess both approach-oriented and problem-solving strategies (“Think of a plan to solve the problem,” “Talk to a friend or family member,” and “Exercise or play sports”) and avoidant coping (“Have a drink,” “Light up a cigarette,” and “Get something to eat”).

#### 2.3.5. Depression

Depression was assessed using the 10-item short version of the Center for Epidemiologic Studies Depression Scale (CESD-10). Cutoff scores for depressive symptoms were greater than or equal to 10. Andresen and colleagues [38] found good predictive accuracy when they compared the CESD-10 to the full-length 20-item version of the CES-D.

#### 2.3.6. PTSD 

PTSD symptom severity was assessed using the PTSD Checklist, Civilian Version (PCLC) [39], a 17-item self-report instrument that asks respondents to rate the extent to which they have been bothered by PTSD symptoms during the previous 30 days using a 5-point scale (1: not at all and 5: extremely). PCLC items parallel DSM-IV PTSD symptom criteria B, C, and D, and a variety of studies support the use of the PCLC as a valid and reliable screening instrument [40–42]. The HRB Survey, and other DoD studies, prefer the PCLC over the military version of the PCL because the military version ignores symptoms from nonmilitary experiences and can miss common causes of deployment or war-related PTSD in women (e.g., sexual assault rather than combat) as well as deployment-related exacerbations of PTSD symptoms if the original inciting trauma is not military related [35]. The standard cutoff was used such that if the sum was greater than or equal to 50, the participant was classified as positive for PTSD; those with scores less than 50 were considered not to have PTSD [39].

#### 2.3.7. Suicidal Ideation and Attempt

Suicidal ideation was assessed by asking respondents about the occurrence of suicidal thoughts within the past year. A dichotomous item indicated any thoughts of suicide versus no thoughts of suicide. This particular item has also been used as a first-level screen for suicidal ideation in previous studies of military personnel [35]. Suicide attempts were assessed by asking respondents whether they had attempted suicide within the past year. A dichotomous item indicated any suicide attempt versus no suicide attempt. Individuals responded based on their own definitions of what it meant to them as having seriously considered or attempted suicide. A third item was created that indicated past year suicide contemplation or attempt versus no suicide contemplation or attempt.

### 2.4. Statistical Analyses

Analyses were conducted using SUDAAN [43] to account for the complex sampling design. Aim 1, the determination of basic prevalence of mental health problems across levels of spirituality and the associations of spirituality with these outcomes, was examined using simple frequencies and crosstabs. Multiple predictor logistic regression models were used to assess the prevalence of mental health outcomes by levels of the key independent variables of spirituality and combat exposure. Aim 2, examination of the moderation or hypothesized buffering effect of greater spirituality on the negative mental health impacts of combat exposure, was addressed with logistic regression models, which included spirituality by combat exposure interaction terms. Significant interaction parameters indicated that the effect of different levels of spirituality was not consistent across levels of combat exposure. For example, although high versus low spirituality may provide modest protection against depression for those not deployed, in the high deployment group the impact could be much greater, with high spirituality personnel showing less combat exposure-related depression relative to their lower spirituality peers. The reference category for spirituality was changed to “high” for models involving interactions because this category was the most dissimilar from the other two and would better highlight differential effects of combat exposure on the mental health outcomes. All regression models included gender, age, race/ethnicity, pay grade, and education as control measures.

## 3. Results

An estimated 30.6% of active duty personnel met screening criteria for depression on the CES-D, 10.7% met screening criteria for PTSD on the PCLC, and 6.0% reported either seriously considering or attempting suicide in the past year (Table 1). As shown in Table 1, 23.1% fell into the high spirituality category, 48.7% fell into the medium spirituality level, and 28.2% fell into the low spirituality level. Lowest rates of all mental health outcomes (except suicide attempts) were observed in the highest spirituality level.

To address the first aim, Table 2 presents the influence of spirituality on mental health outcomes and controlling for demographic and service-related variables. Moderate spirituality had a significant protective effect only for depression. Risk factors for both depression and PTSD were being female, being in younger age groups, non-Hispanic ethnicity, junior-enlisted pay grades, avoidant coping behaviors, and those with moderate or high combat exposure. Results were similar for suicidality with the exception that age and gender effects were not significant. Active coping behaviors were protective for each outcome.

To address the second aim, Table 3 displays the odds ratios and confidence intervals for the logistic models that included combat exposure by spirituality interactions to test the buffering effect of greater spirituality. Four interactions were significant, indicating that the magnitude of the odds ratio for one or more comparisons of spirituality's impact on mental health were conditional on level of combat exposure. To illustrate these differences in patterns, Table 4 shows the adjusted marginal estimates for the mental health outcomes for spirituality levels crossed with the four combat exposure categories. The logistic regression models with interactions effects for combat exposure and spirituality revealed several instances where the impact of combat exposure on mental health was conditional on level of spirituality. Two interactions were significant for spirituality and combat exposure with depression as the dependent variable. Within the low-to-moderate combat exposure group, high spirituality showed a significantly more pronounced buffering effect of exposure on depression. As indicated in Table 4, rates of depression for the high spirituality group within low-moderate combat exposure (21.3%) was considerably lower than the rate in the medium and low spirituality groups (31.1% and 27.3%, resp.). High spirituality was also associated with a more pronounced protective influence against PTSD compared to those with medium spirituality in the low-moderate combat exposure group. (4.4% versus 6.4%). Finally, and counter to expectations, for those in the nondeployed group, medium spirituality was associated with significantly lower likelihood of suicidality relative to those with high spirituality (4.6% versus 6.4%) compared to this relationship in other combat exposure categories.

## 4. Discussion

This study examined the relationship between spirituality, combat exposure, and mental health among a large, population-based sample of active duty military personnel. Overall, spirituality had a positive influence on depression but not on suicidality. This relationship held while controlling for demographic variables, coping behaviors, and combat exposure; however, there was only limited support for the hypothesis that high spirituality would moderate the relationship between combat exposure and mental health outcomes. Findings showed that spirituality did buffer depression and PTSD symptoms but only among those with low-moderate combat exposure. On the other hand, a medium level of spirituality, relative to a high level, was protective of self-reported suicidal ideation/attempt only among those never deployed. This suggests that high levels of spirituality may be associated with greater suicidal ideation or attempt in this nondeployed subgroup of military personnel. It is certainly possible that this subgroup of nondeployed personnel had admitted to suicidal ideation during predeployment screening accounting for their nondeployment. Further research on this group may help elucidate whether this nondeployed group is coming into the service with higher levels of suicidal ideation or whether they are being screened out on mandatory predeployment health assessment forms. This also has implications for chaplains, social workers, and other service providers. Although wounded warriors have captured the attention of the media and have become a priority for service provisions and policy making in the military, these findings suggest that this should not be at the exclusion of those military personnel who have not been deployed.

These results point to the complex relationship between spirituality and mental health, particularly among military personnel. Although consistent with studies that found a relationship between depression and spirituality in various civilian populations, they are not consistent with the Berg [18] study that related spiritual distress with combat-related PTSD among Vietnam veterans, although that study used a different measure of spirituality than the present study. Further, these findings suggest that the buffering role of spirituality in mental health is limited, dependent on type of mental health problem investigated, and may be potentially overwhelmed by great stress, such as high levels of combat exposure. That is, the buffering effect of spirituality may operate within a limited window of stress (e.g., it may not make a difference for those with low amounts of stress or challenges but may be overwhelmed by greater stressors). This effect may also account for studies that have identified negative consequences of high spirituality. That is it may be suggested that high combat exposure may increase emotional vulnerability leading to poorer mental health among highly spiritual personnel. It is clear that spirituality/religiosity plays an important but complex role in the mental health of military personnel and must be investigated within the context of both a wide range of potential covariates and individual mental health indicators or diagnoses. Findings also suggest that it may be beneficial to enhance chaplain involvement across the entire deployment cycle (before, during, and after), ensuring that service members are encouraged to attend to their spiritual needs in whatever manner makes sense to them, and to integrate spiritual assessment into physical and mental health care to improve care of the whole individual. These findings extend those by Edlund et al. [44] using a similar measure who found a strong negative relationship between religiosity and substance abuse in the US population. Given the strong comorbidity of our mental health outcomes with substance abuse, future research should examine the effect of spirituality on mental health controlling for those who abstain from alcohol and other substances on religious grounds.

Among the limitations of this research is the self-report and cross-sectional nature of the data, which limits the ability to make casual inferences. The use of mental health screening measures does not imply clinical diagnoses. Due to the length of the questionnaire, the number of missing items tended to increase slightly with its length. However, the overall response rate was high relative to other military surveys, and most missing items showed less than a 5% missing rate. Although there is limited or a lack of psychometric data on some scales (e.g., coping behaviors scale) used in the survey, all have been utilized successfully in previous military surveys. It should be noted that the definition of spirituality used in this study is an operational one reflecting the specific language of the questionnaire items used to measure what may be thought of as a combination of conceptual definitions. Although religiosity and spirituality are intimately linked, it is widely acknowledged that the two should be studied as distinct concepts in research studies [45]. A large portion of prior research has focused on religiosity (generally used to refer to affiliation with organized and institutional religion) [46], but in recent years there has been an increasing focus on studying the impact of spirituality (generally more personal [2] or broadly defined as an individual's existential relationship with God [47, 48]) on mental health outcomes, specifically depression, anxiety, and suicide. Operational definitions of both constructs have varied widely and often overlap in their assessment [48, 49] as was done in the current study to be consistent with educational materials used in military settings [29]. Unfortunately this combined measure, based on the SAMHSA items, lacks psychometric information. Further studies would benefit from more comprehensive and rigorous measures of spirituality that specified the components of spirituality, such as forgiveness of self and others, and included religion measures, such as religious commitment. Investigating characteristics that differentiate between high, medium, and low levels of spirituality may be particularly informative. Further research into the theory and mechanism by which spirituality is associated with mental health is also warranted.

## 5. Conclusions

In conclusion, the most important finding of this study is that high spirituality appears to have some protective effect for depression and PTSD, but only for those in the low-moderate combat exposure group. This extends and qualifies previous findings of both positive and negative associations between religiosity and mental health outcomes in this and other populations. A number of additional topics for further investigation are identified emphasizing the need for evaluation of potential interventions including spiritual resilience programs implemented by the military.

## Figures and Tables

**Table 1:** Prevalence of mental health outcomes by spirituality level^∗^.

	Overall prevalence (%) *N* = 24, 690	Prevalence among spirituality levels		
		High	Medium	Low
		*N* = 5, 692	*N* = 11, 639	*N* = 6, 102

Depression	30.6	24.9^a^	32.2^b^	32.1^b^
PTSD	10.7	8.8^a^	10.9^b^	11.6^b^
Suicide attempt, past year	2.2	1.8	2.4	2.0
Suicide contemplation, past year	4.6	4.2^a^	4.3^a,b^	5.3^b^
Suicide (attempt or contemplation), past year	6.0	5.5	5.9	6.5

*Excludes missing responses; Ns are unweighted.

^a, b^Estimates for spirituality column differences for depression, PTSD, and suicide contemplation not sharing a common superscript are significantly different at *P* < 0.05.

**Table 2:** Logistic regression model parameters, interactions not included.

Independent variables	DV		
	Depression	PTSD	Suicidality

	OR (95% CI)	OR (95% CI)	OR (95% CI)
Intercept	0.19 (0.14–0.25)^∗^	0.02 (0.01–0.03)^∗^	0.02 (0.02–0.04)^∗^
High spirituality	0.99 (0.90–1.10)	1.16 (0.99–1.37)	1.11 (0.90–1.36)
Moderate spirituality	1.12 (1.01–1.25)^∗^	1.08 (0.91–1.27)	0.96 (0.78–1.17)
Low spirituality	ref	ref	ref
Male	ref	ref	ref
Female	1.64 (1.49–1.79)^∗^	1.63 (1.39–1.91)^∗^	1.24 (1.04–1.47)^∗^
17–20	1.32 (1.10–1.58)^∗^	1.25 (0.94–1.66)	0.98 (0.69–1.39)
21–25	0.96 (0.83–1.12)	0.97 (0.82–1.14)	0.86 (0.67–1.12)
26–34	0.85 (0.74–0.98)^∗^	0.93 (0.76–1.15)	0.79 (0.63–0.99)^∗^
35 or older	ref	ref	ref
Non-Hispanic white	ref	ref	ref
Non-Hispanic black	1.08 (0.98–1.19)	1.01 (0.82–1.24)	1.45 (1.12–1.87)^∗^
Hispanic	1.10 (1.00–1.21)	1.10 (0.90–1.34)	1.46 (1.16–1.83)^∗^
Non-Hispanic other	1.26 (1.10–1.46)^∗^	1.45 (1.21–1.74)^∗^	1.97 (1.56–2.49)^∗^
High school or less	ref	ref	ref
Some college	1.10 (1.00–1.20)^∗^	1.08 (0.93–1.26)	0.96 (0.82–1.12)
College degree or more	1.10 (0.94–1.28)	0.95 (0.76–1.18)	0.98 (0.80–1.21)
E1–E3	1.79 (1.34–2.39)^∗^	3.97 (2.12–7.45)^∗^	2.37 (1.27–4.43)^∗^
E4–E6	1.57 (1.23–2.00)^∗^	2.96 (1.62–5.41)^∗^	2.15 (1.23–3.74)^∗^
E7–E9	1.15 (0.85–1.54)	1.89 (0.97–3.68)	1.68 (0.99–2.84)
W1–W5	0.88 (0.73–1.07)	0.76 (0.17–3.51)	1.71 (0.90–3.24)
O1–O3	1.13 (0.89–1.42)	1.88 (0.89–3.97)	1.69 (0.96–2.98)
O4–O10	ref	ref	ref
Not deployed	0.99 (0.87–1.13)	1.12 (0.91–1.38)	1.08 (0.89–1.31)
No combat exposure	ref	ref	ref
Moderate combat exposure	1.14 (1.04–1.26)^∗^	1.11 (0.88–1.39)	0.84 (0.74–0.96)^∗^
High combat exposure	1.52 (1.35–1.72)^∗^	3.57 (3.00–4.26)^∗^	1.28 (1.06–1.56)^∗^
Active coping	0.68 (0.65–0.72)^∗^	0.67 (0.62–0.73)^∗^	0.65 (0.58–0.73)^∗^
Avoidant coping	3.21 (3.02–3.41)^∗^	3.10 (2.80–3.43)^∗^	1.90 (1.70–2.12)^∗^

**Table 3:** Logistic regression model parameters, interactions included.

Independent variables	DV		
	Depression	PTSD	Suicidality

	OR (95% CI)	OR (95% CI)	OR (95% CI)
High spirituality	ref	ref	ref
Moderate spirituality	1.07 (0.91–1.27)	0.73 (0.51–1.02)	1.27 (0.85–1.89)
Low spirituality	0.98 (0.85–1.13)	0.81 (0.52–1.27)	1.06 (0.68–1.65)
Male	ref	ref	ref
Female	1.63 (1.49–1.79)^∗^	1.63 (1.40–1.90)^∗^	1.24 (1.04–1.47)^∗^
17–20	1.33 (1.11–1.60)^∗^	1.25 (0.94–1.67)	0.98 (0.69–1.40)
21–25	0.96 (0.83–1.11)	0.97 (0.82–1.14)	0.87 (0.67–1.12)
26–34	0.85 (0.74–0.97)^∗^	0.93 (0.76–1.15)	0.79 (0.63–0.99)^∗^
35 or older	ref	ref	ref
Non-Hispanic white	ref	ref	ref
Non-Hispanic black	1.08 (0.98–1.19)	1.01 (0.82–1.24)	1.44 (1.12–1.86)^∗^
Hispanic	1.10 (0.99–1.21)	1.10 (0.90–1.33)	1.46 (1.17–1.83)^∗^
Non-Hispanic other	1.27 (1.10–1.46)^∗^	1.46 (1.22–1.75)^∗^	1.97 (1.57–2.49)^∗^
High school or less	ref	ref	ref
Some college	1.10 (1.00–1.20)^∗^	1.08 (0.93–1.26)	0.96 (0.82–1.12)
College degree or more	1.10 (0.95–1.28)	0.95 (0.76–1.18)	0.99 (0.80–1.21)
E1–E3	1.79 (1.33–2.40)^∗^	3.95 (2.10–7.42)^∗^	2.39 (1.28–4.47)^∗^
E4–E6	1.55 (1.21–1.99)^∗^	2.92 (1.59–5.37)^∗^	2.16 (1.24–3.74)^∗^
E7–E9	1.14 (0.84–1.54)	1.87 (0.95–3.66)	1.68 (1.00–2.84)
W1–W5	0.87 (0.71–1.06)	0.75 (0.16–3.50)	1.70 (0.89–3.23)
O1–O3	1.11 (0.88–1.40)	1.85 (0.88–3.93)	1.69 (0.96–2.97)
O4–O10	ref	ref	ref
Not deployed	1.18 (0.97–1.42)	1.14 (0.76–1.69)	1.56 (1.08–2.26)^∗^
No combat exposure	ref	ref	ref
Moderate combat exposure	0.83 (0.65–1.05)	0.69 (0.44–1.07)	1.08 (0.66–1.75)
High combat exposure	1.42 (1.14–1.77)^∗^	2.97 (1.94–4.56)^∗^	1.64 (0.97–2.80)
Active coping	0.68 (0.65–0.72)^∗^	0.67 (0.62–0.73)^∗^	0.65 (0.58–0.73)^∗^
Avoidant coping	3.21 (3.02–3.41)^∗^	3.10 (2.80–3.43)^∗^	1.89 (1.70–2.11)^∗^
High spirituality, nondeployed	NA	NA	NA
High spirituality, no exposure	NA	NA	NA
High spirituality, moderate exposure	NA	NA	NA
High spirituality, high exposure	NA	NA	NA
Medium spirituality, nondeployed	0.81 (0.65–1.01)	1.01 (0.65–1.58)	0.55 (0.35–0.87)^∗^
Medium spirituality, no exposure	NA	NA	NA
Medium spirituality, moderate exposure	1.55 (1.20–2.01)^∗^	2.05 (1.22–3.43)^∗^	0.70 (0.37–1.30)
Medium spirituality, high exposure	1.09 (0.84–1.41)	1.41 (0.86–2.31)	0.65 (0.34–1.24)
Low spirituality, nondeployed	0.80 (0.63–1.01)	0.93 (0.54–1.61)	0.77 (0.48–1.24)
Low spirituality, no exposure	NA	NA	NA
Low spirituality, moderate exposure	1.42 (1.07–1.88)^∗^	1.46 (0.81–2.65)	0.81 (0.46–1.43)
Low spirituality, high exposure	1.10 (0.78–1.56)	1.06 (0.57–2.00)	0.89 (0.42–1.89)

Note: NA indicates interaction term included reference level of one or more predictors and was not estimable.

**Table 4:** Adjusted estimates of spirituality by combat exposure.

Combat exposure	Not deployed			No exposure			Low-moderate exposure			High exposure		
Spirituality	High	Med	Low	High	Med	Low	High	Med	Low	High	Med	Low
Depression	27.8	25.1	23.1	24.7	26.0	24.2	21.3	31.1	27.3	31.7	35.2	33.3
PTSD	7.1	5.3	5.5	6.3	4.7	5.2	4.4	6.4	5.2	16.7	17.0	14.8
Suicidality	6.4	4.6	5.3	4.2	5.3	4.5	4.5	4.0	3.9	6.8	6.7	6.4
